# Effects of dietary protein/energy ratio on growth performance, carcass trait, meat quality, and plasma metabolites in pigs of different genotypes

**DOI:** 10.1186/s40104-015-0036-x

**Published:** 2015-08-15

**Authors:** Yingying Liu, Xiangfeng Kong, Guoli Jiang, Bi’e Tan, Jinping Deng, Xiaojian Yang, Fengna Li, Xia Xiong, Yulong Yin

**Affiliations:** Key Laboratory of Agro-ecological Processes in Subtropical Region, Hunan Provincial Engineering Research Center of Healthy Livestock and Poultry, and Scientific Observing and Experimental Station of Animal Nutrition and Feed Science in South-Central, Ministry of Agriculture, Institute of Subtropical Agriculture, Chinese Academy of Sciences, Changsha, Hunan 410125 China; Hunan Animal Science and Veterinary Medicine Research Institute, Changsha, Hunan 410131 China; College of Animal Science and Technology, Hunan Agricultural University, Changsha, Hunan 410128 China; Southern Research and Outreach Center, University of Minnesota, Waseca, MN 56093 USA; University of Chinese Academy of Sciences, Beijing, 100049 China

**Keywords:** Dietary protein/energy ratio, Growth performance, Meat quality, Mini-pig, Plasma metabolites

## Abstract

**Background:**

The protein/energy ratio is important for the production performance and utilization of available feed resources by animals. Increased protein consumption by mammals leads to elevated feed costs and increased nitrogen release into the environment. This study aimed to evaluate the effects of dietary protein/energy ratio on the growth performance, carcass traits, meat quality, and plasma metabolites of pigs of different genotypes.

**Methods:**

Bama mini-pigs and Landrace pigs were randomly assigned to two dietary treatment groups (Chinese conventional diet with low protein/energy ratio or National Research Council diet with high protein/energy ratio; n = 24 per treatment) in a 2 × 2 factorial arrangement. Blood and muscle samples were collected at the end of the nursery, growing, and finishing phases.

**Results:**

We observed significant interactions (*P* < 0.05) between breed and diet for total fat percentage, intramuscular fat (IMF) content, protein content in *biceps femoris* (BF) muscle, and plasma urea nitrogen (UN) concentration in the nursery phase; for average daily gain (ADG), average daily feed intake (ADFI), dry matter, IMF content in *psoas major* (PM) muscle, and plasma total protein and albumin concentrations in the growing phase; and for drip loss and plasma UN concentration in the finishing phase. Breed influenced (*P* < 0.05) growth performance, carcass traits, and meat quality, but not plasma metabolites. Throughout the trial, Landrace pigs showed significantly higher (*P* < 0.05) ADG, ADFI, dressing percentage, lean mass rate, and loin-eye area than did Bama mini-pigs, but significantly lower (*P* < 0.05) feed/gain ratio, fat percentage, backfat thickness, and IMF content. Dietary protein/energy ratio influenced the pH value, chemical composition of BF and PM muscles, and plasma activities of glutamic-pyruvic transaminase and gamma-glutamyl transpeptidase, and plasma concentration of UN.

**Conclusions:**

Compared with Landrace pigs, Bama mini-pigs showed slower growth and lower carcass performance, but had better meat quality. Moreover, unlike Landrace pigs, the dietary protein/energy ratio did not affect the growth performance of Bama mini-pigs. These results suggest that, in swine production, low dietary protein/energy ratio may be useful for reducing feed costs and minimizing the adverse effects of ammonia release into the environment.

**Electronic supplementary material:**

The online version of this article (doi:10.1186/s40104-015-0036-x) contains supplementary material, which is available to authorized users.

## Background

The pig is one of the most economically important species among domesticated livestock and is a major protein source for human consumption. A major objective of pig production is to increase skeletal muscle growth and reduce excess fat accretion. Livestock producers use nutritional modifiers in an attempt to increase protein accretion in the muscle, while they often simultaneously reduce fat deposition [1, 2]. The pattern of fat deposition in pigs in the growing-finishing phase affects carcass and meat quality [3]. Genetic selection of pigs for leaner meat has also resulted in reduced intramuscular fat (IMF) content, and consumers perceive the meat thus obtained as tougher, less moist, and poorly flavored. Thus, the livestock industry faces the challenge of increasing the IMF content of pork so that consumers may have a satisfactory experience, while simultaneously producing minimal visible fat, which is a deterrent to health-conscious consumers.

In China, several rich resources of indigenous pig breeds are available, with more than 30 breeds established. In addition to their economic significance, pigs, particularly miniature pigs (mini-pigs), are considered important animal models for human disease and xenotransplantation research because of their physiological and anatomical similarities to humans [4, 5]. Bama mini-pigs (*Sus scrofa domestica*), a Chinese indigenous mini-pig breed which originated in Bama County, Guangxi Province, is a promising animal model [6, 7] with an obese genotype. In contrast, Landrace, a representative lean genotype, is fast-growing breed and produces a relatively large amount of meat, and it is therefore more attractive to producers. Although muscle growth and meat quality considerably differ between Western and indigenous Chinese pig breeds, it is not clear how nutrients mediate the effect of genetic background on animal growth and meat quality. The growth and development processes of pigs, which involve changes in body weight (BW) and shape as well as metabolic and physiological functions, depend on factors such as the breed, nutritional status, and feeding condition of the animal. Against this background, the objective of the present study was to examine the effects of dietary protein/energy ratio on the growth performance, muscle development, and plasma metabolites (which are indicators of nitrogen metabolism) in Bama mini-pigs and Landrace pigs at different phases of growth.

## Methods

### Study animals

The experiment was carried out in accordance with the Chinese guidelines for animal welfare and experimental protocol, and was approved by the Animal Care and Use Committee of the Institute of Subtropical Agriculture, Chinese Academy of Sciences [8]. Ninety-six barrows (including 48 Bama mini-pigs, a Chinese local breed [average initial BW, 3.38 ± 0.96 kg], and 48 Landrace piglets [average initial BW, 7.68 ± 0.89 kg]) were used in this study.

### Study design

The experiment was a 2 × 2 factorial arrangement, with two breeds (Bama mini-pig *vs.* Landrace) and two dietary treatments (Chinese conventional diet [GB diet] and National Research Council [NRC] diet), resulting in a total of four treatments (Table 1). Piglets of each breed were randomly assigned to one of two dietary treatment groups (n = 24 per treatment). The NRC diet was formulated to meet the nutrient requirements recommended by NRC (2012) [9] and had a high protein/energy ratio, whereas the GB diet was formulated per the recommendations of Chinese National Feeding Standard for Swine (GB, 2004) [10] and had a low protein/energy ratio (Table 2 and Additional file 1: Table S1). The animals were individually housed in 0.6 m × 1.2 m pens with hard plastic slatted flooring. Each pen was equipped with a stainless steel feeder and a nipple drinker. The animals had free access to drinking water and feed. The room temperature was maintained at 25–27 °C. All pigs were fed three times per day at 0800, 1300, and 1800 h. Dietary phase changes were noted on the day on which the pigs were weighed; these changes were noted on the same day for all treatment types. Every 2 weeks, feed intake was recorded in order to determine average daily gain (ADG), average daily feed intake (ADFI), and the feed intake to body gain ratio (F/G).

**Table 1:** Animals and treatments

Item	Landrace pig			Bama mini-pig		
	Body weight	GB diet group	NRC diet group	Body weight	GB diet group	NRC diet group
Nursery phase	10–20, kg	GB diet 1	NRC diet 1	8–15, kg	GB diet 1	NRC diet 1
Growing phase	20–50, kg	GB diet 2	NRC diet 2	15–35, kg	GB diet 2	NRC diet 2
Finishing phase	50–90, kg	GB diet 3	NRC diet 3	35–55, kg	GB diet 3	NRC diet 3

GB diet, Chinese conventional diet

**Table 2:** Nutrient content of experimental diets

Item	NRC diet 1	NRC diet 2	NRC diet 3	GB diet 1	GB diet 2	GB diet 3
Digestible energy, MJ/kg	14.22	14.21	14.22	13.46	13.40	13.40
Crude protein, %	20.06	18.01	15.11	18.03	16.05	13.46
Protein/energy ratio	1.41	1.27	1.06	1.34	1.20	1.00

GB diet, Chinese conventional diet

### Sample collection

According to the feeding standards, the BW range for nursery, growing, and finishing phases (ending with market weight) was defined as 8–20, 20–50, and 50–90 kg, respectively, for Landrace pigs, and 3–15, 15–35, and 35–55 kg, respectively, for Bama mini-pig (Table 1). Nursery, growing, and finishing phases comprised feeding for 39, 44 and 30 days, respectively, for Landrace pigs, and 55, 48 and 21 days, respectively, for Bama mini-pigs. At the end of each phase (i.e., when BW reached 20, 50, and 90 kg for Landrace pigs, and 15, 35, and 55 kg for Bama mini-pigs), eight pigs from each treatment were randomly weighed, bled, and sacrificed for the evaluation of carcass characteristics and meat quality. Briefly, the animals were fasted for 12 h to avoid the effect of feed intake on postprandial biochemical measurements and then pre-slaughter BW was measured. Thereafter, blood samples were collected into 10-mL centrifuge tubes containing sodium heparin (14.3 USP units/mL). Next, the pigs were held under general anesthesia and sacrificed by a jugular vein injection of 4 % sodium pentobarbital solution (40 mg/kg BW) [11]. After the head, legs, tail, and viscera were removed, the carcass was split longitudinally. *Longissimus dorsi* (LD), *biceps femoris* (BF), and *psoas major* (PM) muscles from the right-side of each carcass were immediately sampled after slaughter, and stored at −20 °C for biochemical analysis (including evaluation of dry matter [DM], crude protein [CP], and crude lipid contents). The LD muscle on the right-side carcass was removed, and approximately 2.5-cm-thick sections were cut from the anterior end for assessment of meat quality before and after chilling the carcass for 24 h [2]. Blood samples were subsequently centrifuged at 900 × *g* for 10 min at 4 °C to recover plasma. Plasma samples were stored at −80 °C until analysis.

### Determination of carcass composition

Pre-slaughter BW, carcass weight, carcass length, backfat thickness, and loin-eye area at the 10^th^ rib were measured immediately *post-mortem* according to the Chinese Guidelines on Performance Measurement Technology and Regulations for Pigs [12]. Carcass straight length was measured from the first rib to the end of the pubic bone. Backfat thickness was measured using a vernier caliper, and the average measurements at three points: the first rib, last rib, and last lumbar vertebra were recorded. The left side of each carcass was weighed and then physically dissected into skin, skeletal muscle, fat, and bone for evaluation of carcass characteristics. These components were weighed, and the weights were multiplied by 2 to calculate the percentage of the whole carcass that each component constituted. Dressing percentage was calculated as carcass weight divided by live BW.

### Assessment of meat quality

Meat quality was examined at the end of experiment by determining the pH, muscle color, drip loss, and cooking yield. Initial pH (pH_45min_) and final pH (pH_24h_) values were measured in triplicate at the 6^th^ to 7^th^ rib position at 45 min and 24-h postmortem, respectively, using a hand-held pH meter (Russell CD700; Russell pH Limited, Germany). Muscle color scores were assigned to LD muscle at the 10^th^ rib interface by using a Konica Minolta chromameter (CR410; Konica Minolta Sensing, Inc., Tokyo, Japan) with an 8-mm measuring port, D65 illuminant, and 10 observers. Hunter lightness (L^*^), redness (a^*^), and yellowness (b^*^) values were recorded in triplicate. For evaluating drip loss, on the day of slaughter, approximately 100 g fresh LD muscle was weighed and placed in a Whirl-Pak bag, suspended in a 4 °C cooler for 24 h, reweighed, and drip loss was recorded. Percentage of cooking meat was measured by determining the weight of cooked LD muscle. The muscle sample was weighed and covered in a container before cooking. Immediately after cooking for 45 min at 100 °C, the sample was removed from the container and dried with a paper towel, then reweighed. Cooking yield was expressed using the following formula: Cooking yield = (cooked weight/raw weight) × 100.

### Chemical analysis of skeletal muscle

Chemical composition of the skeletal muscle was analyzed in duplicate according to AOAC methods (1997) [13]. DM content of muscle was determined gravimetrically by oven drying the samples at 110 °C for 24 h. CP and lipid contents were measured using Kjeldahl and Soxhlet extraction methods, respectively [2].

### Analysis of plasma metabolites

Plasma activities of alkaline phosphatase (ALP), glutamic-pyruvic transaminase (GPT), glutamic-oxaloacetic transaminase (GOT), lactate dehydrogenase (LDH), creatine phosphokinase (CPK), and gamma-glutamyl transpeptidase (GGT), and plasma concentrations of albumin (Alb), total protein (TP), ammonia (AMM), and urea nitrogen (UN) were analyzed using a CX-4 Automatic Biochemical Analyzer (Beckman Inc., USA) and commercial kits (Leadman Biochemistry Technology Company, Beijing, China) according to the manufacturers’ instructions [14].

### Statistical analysis

Data were analyzed by a mixed-effects model using the SAS version 8.2 (SAS Institute Inc., Cary, NC, USA). Diet, breed, and their interactions were included in the statistical model. Effects were considered statistically significant at *P* < 0.05. Probability values between 0.05 and 0.10 were considered to be trends.

## Results

### Growth performance

All pigs showed healthy growth throughout the experimental period. ADG and ADFI of Bama mini-pigs were lower (*P* < 0.05), whereas F/G was higher (*P* < 0.05) when compared with Landrace pigs in the same phase and fed the same diet (Table 3). The growth performance of Bama mini-pigs did not significantly differ (*P* > 0.05) between dietary treatments in any of the three phases. However, in the growing phase, Landrace pigs fed the GB diet had higher (*P* < 0.05) ADG and ADFI than those fed the NRC diet, indicating breed × diet interactions (*P* < 0.05). In contrast, in the finishing phase, Landrace pigs fed the GB diet had lower (*P* < 0.05) ADG than those fed the NRC diet.

**Table 3:** Effects of dietary protein/energy ratio and breed on growth performance of pigs

Item	Landrace pig		Bama mini-pig		SEM	*P-*value		
	GB diets	NRC diets	GB diets	NRC diets		Breed	Diet	B × D
Nursery phase (*n* = 24)								
Initial BW, kg	7.71^a^	7.64^a^	3.37^b^	3.38^b^	0.19	<0.001	0.88	0.83
Final BW, kg	22.95^a^	23.06^a^	17.29^b^	17.91^b^	0.81	<0.001	0.66	0.76
Average daily gain, g	390.80^a^	395.40^a^	253.10^b^	264.10^b^	15.36	<0.001	0.62	0.84
Average daily feed intake, kg	0.76^a^	0.71^a^	0.62^b^	0.63^b^	0.03	<0.001	0.42	0.41
Feed to gain ratio	1.97^b^	1.87^b^	2.53^a^	2.38^a^	0.07	<0.001	0.09	0.73
Growing phase (*n* = 16)								
Initial BW, kg	24.74^a^	25.15^a^	20.55^b^	19.66^b^	0.66	<0.001	0.73	0.34
Final BW, kg	61.82^a^	56.37^b^	38.81^c^	38.95^c^	1.70	<0.001	0.13	0.12
Average daily gain, g	842.80^a^	709.60^b^	380.60^c^	401.80^c^	31.39	<0.001	0.09	0.02
Average daily feed intake, kg	1.96^a^	1.64^b^	1.79^b^	1.68^b^	0.05	0.23	<0.001	0.04
Feed to gain ratio	2.33^a^	2.43^b^	4.77^a^	4.65^a^	0.27	<0.001	0.98	0.70
Finishing phase (*n* = 8)								
Initial BW, kg	65.03^a^	62.83^a^	44.07^b^	45.82^b^	1.07	<0.001	0.87	0.15
Final BW, kg	91.03^a^	92.03^a^	51.40^b^	52.76^b^	1.46	<0.001	0.52	0.92
Average daily gain, g	866.70^b^	973.30^a^	348.80^c^	330.50^c^	41.87	<0.001	0.40	0.24
Average daily feed intake, kg	3.12^a^	3.07^a^	1.69^b^	1.83^b^	0.06	<0.001	0.59	0.22
Feed to gain ratio	3.66^b^	3.19^b^	5.62^a^	5.79^a^	0.49	0.001	0.80	0.61

^a, b, c^ Mean values with unlike superscript letters were significantly different (P < 0.05)

GB diets, Chinese conventional diets; B × D, breed × diet interaction

### Carcass quality

Table 4 shows the effects of treatments on carcass characteristics. In all phases, Bama mini-pigs had significantly lower (*P* < 0.05) dressing percentage, carcass length, loin-eye area, and skeletal muscle percentage (15–25 units difference), but higher (*P* < 0.05) total fat percentage (1–20 units difference) than did Landrace pigs. Except during the nursery phase, the backfat thickness of Bama mini-pigs was greater (*P* < 0.05) than that of Landrace pigs (2- to 5-fold difference after the nursery phase). In the nursery and finishing phases, Bama mini-pigs fed the NRC diet showed significantly higher (*P* < 0.05) dressing percentages than those fed the GB diet. In the nursery phase, Landrace pigs fed the NRC diet had significantly greater (*P* < 0.05) loin-eye area and reduced backfat thickness (*P* < 0.05) than those fed the GB diet. However, in the growing phase, Landrace pigs fed the GB diet had longer carcasses, more total skeletal muscle, and lower percentage of fat than those fed the NRC diet (*P* < 0.05).

**Table 4:** Effects of dietary protein/energy ratio and breed on carcass performance in pigs

Item	Landrace pig		Bama mini-pig		SEM	*P-*value		
	GB diets	NRC diets	GB diets	NRC diets		Breed	Diet	B × D
Nursery phase								
Pre-slaughter BW, kg	19.39^a^	19.15^a^	12.00^b^	14.40^b^	0.84	<0.001	0.21	0.13
Left carcass BW, kg	6.25^a^	6.17^a^	3.20^c^	4.19^b^	0.32	<0.001	0.16	0.10
Dressing percentage, %	64.29^a^	64.15^a^	53.26^c^	58.16^b^	1.32	<0.001	0.08	0.06
Carcass length (bevel), cm	25.69^a^	23.76^a^	14.92^b^	15.69^b^	1.13	<0.001	0.61	0.24
Carcass length (straight), cm	24.04^a^	21.72^a^	13.56^b^	14.35^b^	1.03	<0.001	0.46	0.14
Backfat thickness, mm	7.78^a^	5.64^b^	6.81^ab^	7.19^ab^	0.52	0.75	0.78	0.14
Loin-eye area, mm^2^	904.40^b^	1,124.20^a^	372.30^c^	437.80^c^	73.41	<0.001	0.06	0.30
Total skeletal muscle, %	61.23^a^	65.75^a^	47.70^b^	46.38^b^	1.58	<0.001	0.32	0.07
Total fat, %	6.47^b^	3.63^b^	19.36^a^	23.54^a^	1.56	<0.001	0.67	0.03
Growing phase								
Pre-slaughter BW, kg	58.51^a^	49.21^b^	33.62^c^	34.36^c^	1.87	<0.001	0.05	0.02
Left carcass BW, kg	20.98^a^	17.66^b^	10.91^c^	11.23^c^	0.71	<0.001	0.07	0.03
Dressing percentage, %	71.67^a^	71.74^a^	65.04^b^	65.13^b^	0.97	<0.001	0.94	0.99
Carcass length (bevel), cm	85.21^a^	80.57^b^	69.33^c^	69.29^c^	1.18	<0.001	0.08	0.08
Carcass length (straight), cm	82.07^a^	76.57^b^	59.00^c^	61.29^c^	1.12	<0.001	0.20	0.004
Backfat thickness, mm	6.07^b^	6.54^b^	30.70^a^	33.36^a^	1.33	<0.001	0.29	0.46
Loin-eye area, mm^2^	1,919.70^a^	1,724.20^a^	696.70^b^	671.70^b^	120.20	<0.001	0.41	0.52
Total skeletal muscle, %	66.53^a^	62.97^b^	40.70^c^	40.08^c^	1.08	<0.001	0.09	0.22
Total fat, %	10.07^c^	13.45^b^	34.60^a^	35.88^a^	0.87	<0.001	0.02	0.28
Finishing phase								
Pre-slaughter BW, kg	86.79^a^	92.03^a^	49.84^b^	49.19^b^	2.15	<0.001	0.36	0.24
Left carcass BW, kg	32.39^a^	34.79^a^	17.14^b^	17.69^b^	0.90	<0.001	0.16	0.38
Dressing percentage, %	74.50^a^	75.61^a^	68.91^c^	71.90^b^	0.76	<0.001	0.03	0.29
Carcass length (bevel), cm	92.29^a^	92.00^a^	81.80^b^	78.86^b^	1.32	<0.001	0.30	0.39
Carcass length (straight), cm	87.40^a^	87.83^a^	79.90^b^	77.00^b^	1.22	<0.001	0.39	0.25
Backfat thickness, mm	22.69^b^	23.52^b^	40.40^a^	43.29^a^	1.42	<0.001	0.27	0.53
Loin-eye area, mm^2^	3,303.90^a^	2,836.60^a^	1,018.50^b^	812.40^b^	155.78	<0.001	0.07	0.47
Total skeletal muscle, %	61.84^a^	62.73^a^	37.40^b^	38.38^b^	0.97	<0.001	0.41	0.98
Total fat, %	15.65^b^	16.81^b^	38.00^a^	37.85^a^	1.01	<0.001	0.67	0.57

^a, b, c^ Mean values with unlike superscript letters were significantly different (*P* < 0.05). *n =* 8

GB diets, Chinese conventional diets; B × D, breed × diet interaction

### Meat quality

Compared with Landrace pigs, Bama mini-pigs had lower (*P* < 0.05) pH_45min_ and pH_24h_, but higher (*P* < 0.05) a^*^ and cooking yield (Table 5). For Bama mini-pigs, the NRC diet showed increased (*P* < 0.05) pH_24h_ in the finishing phase. A breed × diet interaction (*P* < 0.05) was observed for dripping loss.

**Table 5:** Effects of dietary protein/energy ratio and breed on meat quality in finishing pigs

Item	Landrace pig		Bama mini-pig		SEM	*P-*value		
	GB diets	NRC diets	GB diets	NRC diets		Breed	Diet	B × D
pH value (45 min)	6.38^a^	6.22^ab^	6.08^b^	6.08^b^	0.07	0.01	0.34	0.36
pH value (24 h)	5.45^a^	5.49^a^	5.29^b^	5.45^a^	0.04	0.06	0.05	0.27
L*	47.62	47.47	48.55	48.11	1.00	0.85	0.27	0.33
a*	12.87^c^	13.80^bc^	14.30^ab^	15.29^a^	0.30	<0.001	0.01	0.94
b*	5.62	5.68	6.11	5.70	0.39	0.57	0.69	0.60
Drip loss, %	1.99^a^	1.53^ab^	1.16^b^	1.62^ab^	0.20	0.12	0.99	0.05
Cooking yield, %	53.45	53.22	55.49	55.78	0.81	0.02	0.97	0.78

^a, b, c^Mean values with unlike superscript letters were significantly different (*P* < 0.05). *n =* 8

GB diets, Chinese conventional diets; B × D, breed × diet interaction

### Chemical composition of muscle

Chemical compositions of LD, BF, and PM muscles were examined (Table 6). The lipid content of these muscles was significantly higher (*P* < 0.05) in Bama mini-pigs than in Landrace pigs. The DM contents of BF in the nursery phase; that of LD, BF, and PM in the growing phase; and that of LD and BF in the finishing phase were higher (*P* < 0.05) in Bama mini-pigs than in Landrace pigs. In addition, compared with the Landrace pigs, Bama mini-pigs had higher (*P* < 0.05) CP levels in the BF and PM muscles in the nursery phase and in the LD muscle in the growing phase. Bama mini-pigs fed the GB diet had higher (*P* < 0.05) lipid content in the BF muscle in the nursery phase, and significantly higher (*P* < 0.05) contents of DM, lipid, and CP in the BF and PM muscles in the growing phase, than those fed the NRC diet. In the finishing phase, Bama mini-pigs fed the GB diet had lower (*P* < 0.05) contents of DM, lipid, and CP in the BF muscle than those fed the NRC diet.

**Table 6:** Effects of dietary protein/energy ratio and breed on muscle chemical composition in pigs, %

Item	Landrace pig		Bama mini-pig		SEM	*P-*value		
	GB diets	NRC diets	GB diets	NRC diets		Breed	Diet	B × D
Nursery phase								
* Longissimus dorsi* muscle								
Dry matter	23.75	23.68	23.61	24.09	0.33	0.69	0.55	0.41
Intramuscular fat	1.22^b^	1.06^b^	2.82^a^	3.54^a^	0.26	<0.001	0.30	0.10
Crude protein	18.34^b^	19.69^a^	19.47^a^	19.61^a^	0.36	0.17	0.05	0.11
* Biceps femoris* muscle								
Dry matter	22.11^bc^	21.39^c^	23.39^a^	23.15^ab^	0.42	0.001	0.26	0.57
Intramuscular fat	0.86^c^	0.99^c^	3.25^a^	2.45^b^	0.22	<0.001	0.14	0.05
Crude protein	17.60^bc^	16.49^c^	18.51^ab^	19.01^a^	0.40	<0.001	0.46	0.05
* Psoas major* muscle								
Dry matter	22.21	22.42	21.77	22.37	0.35	0.50	0.27	0.59
Intramuscular fat	0.89^b^	0.94^b^	2.48^a^	2.32^a^	0.20	<0.001	0.78	0.62
Crude protein	16.94^b^	17.41^b^	17.72^ab^	18.56^a^	0.35	0.01	0.09	0.63
Growing phase								
* Longissimus dorsi* muscle								
Dry matter	25.07^b^	25.05^b^	29.11^a^	27.37^a^	0.63	<0.001	0.24	0.25
Intramuscular fat	1.16^b^	1.50^b^	3.73^a^	3.50^a^	0.34	<0.001	0.89	0.48
Crude protein	21.27^b^	20.77^b^	21.98^ab^	22.73^a^	0.39	0.007	0.79	0.18
* Biceps femoris* muscle								
Dry matter	23.15^b^	22.89^b^	26.79^a^	23.76^b^	0.75	0.01	0.05	0.10
Intramuscular fat	1.01^b^	0.81^b^	2.77^a^	1.68^b^	0.30	<0.001	0.05	0.17
Crude protein	19.80^b^	19.24^b^	22.13^a^	19.35^b^	0.71	0.13	0.04	0.16
* Psoas major* muscle								
Dry matter	23.67^c^	23.68^c^	26.78^a^	24.97^b^	0.36	<0.001	0.04	0.03
Intramuscular fat	1.06^b^	1.19^b^	4.66^a^	2.25^b^	0.36	<0.001	0.01	0.005
Crude protein	19.82	20.17	21.07	20.84	0.51	0.10	0.91	0.62
Finishing phase								
* Longissimus dorsi* muscle								
Dry matter	26.16^c^	26.56^bc^	28.74^a^	28.26^ab^	0.58	0.004	0.95	0.50
Intramuscular fat	2.59^b^	2.48^b^	5.85^a^	4.50^a^	0.44	<0.001	0.15	0.22
Crude protein	21.32	22.02	21.73	21.93	0.47	0.76	0.40	0.64
* Biceps femoris* muscle								
Dry matter	24.97^b^	25.18^b^	25.43^b^	28.93^a^	0.77	0.02	0.04	0.07
Intramuscular fat	1.42^c^	1.88^c^	3.65^b^	5.23^a^	0.31	<0.001	0.01	0.12
Crude protein	20.84^ab^	21.19^ab^	19.41^b^	22.85^a^	0.81	0.90	0.05	0.10
* Psoas major* muscle								
Dry matter	25.47	24.70	26.42	26.04	0.57	0.10	0.40	0.77
Intramuscular fat	2.46^b^	2.43^b^	4.01^a^	4.00^a^	0.38	0.002	0.96	0.97
Crude protein	21.08	21.30	20.70	20.91	0.48	0.51	0.71	0.99

^a, b, c^Mean values with unlike superscript letters were significantly different (*P* < 0.05). *n =* 8

GB diets, Chinese conventional diets; B × D, breed × diet interaction

### Plasma metabolites

Plasma biochemical analytes of Bama mini-pigs and Landrace pigs are listed in Table 7. In the nursery phase, no breed × diet interactions were observed (*P* > 0.05) for plasma biochemical analytes except for UN (*P* = 0.05). For pigs fed the GB diet, the plasma ALP activity was lower (*P* < 0.05), but UN concentration was higher (*P* < 0.05) in Landrace pigs than in Bama mini-pigs. Within each breed, diet did not influence (*P* > 0.05) the concentrations of plasma metabolites.

**Table 7:** Effects of dietary protein/energy ratio and breed on the plasma biochemical parameters in pigs

Item	Landrace		Bama mini-pig		SEM	*P-*value		
	GB diets	NRC diets	GB diets	NRC diets		Breed	Diet	B × D
Nursery phase								
ALP, U/L	173.63^b^	148.88^b^	235.88^a^	182.63^ab^	18.64	0.01	0.04	0.45
GPT, U/L	62.88	77.75	67.63	81.00	7.94	0.62	0.08	0.92
GOT, U/L	64.00	89.00	71.50	101.63	14.75	0.50	0.07	0.86
LDH, U/L	665.50	742.63	654.63	793.00	60.15	0.74	0.08	0.61
CPK, U/L	1,622.00	2,218.50	1,654.40	1,901.00	347.96	0.68	0.23	0.62
GGT, U/L	111.00	75.38	66.00	82.63	16.63	0.27	0.57	0.13
Alb, g/L	31.53	32.31	34.97	34.74	2.14	0.18	0.89	0.81
TP, g/L	61.92^b^	68.39^ab^	64.67^ab^	71.51^a^	2.72	0.29	0.02	0.94
AMM, μmol/L	213.31	113.75	167.10	129.14	34.25	0.66	0.05	0.38
UN, mmol/L	6.79^a^	5.86^ab^	5.06^b^	5.81^ab^	0.41	0.04	0.82	0.05
Growing phase								
ALP, U/L	207.67^a^	192.33^a^	123.67^b^	90.75^b^	18.00	<0.001	0.24	0.67
GPT, U/L	99.67^a^	71.33^b^	69.50^b^	72.13^b^	4.76	0.03	0.31	0.17
GOT, U/L	92.67	84.83	64.83	63.38	16.26	0.19	0.80	0.86
LDH, U/L	854.21^a^	996.22^a^	490.50^b^	503.44^b^	116.31	0.003	0.56	0.62
CPK, U/L	1,614.50	1,640.00	1,826.30	1,608.00	337.95	0.81	0.80	0.75
GGT, U/L	120.00^a^	71.00^b^	67.33^b^	72.00^b^	13.04	0.18	0.26	0.16
Alb, g/L	48.98^a^	39.35^b^	38.65^b^	37.75^b^	1.73	0.005	0.01	0.03
TP, g/L	87.28^a^	69.82^b^	84.80^a^	85.02^a^	3.52	0.12	0.04	0.03
AMM, μmol/L	88.02	112.68	96.18	139.16	11.63	0.32	0.06	0.60
UN, mmol/L	6.52	6.56	5.25	5.42	0.68	0.13	0.89	0.93
Finishing phase								
ALP, U/L	114.00^ab^	157.83^a^	85.60^b^	117.14^ab^	13.98	0.05	0.03	0.72
GPT, U/L	62.80^ab^	70.00^a^	51.60^b^	67.14^ab^	4.70	0.22	0.05	0.46
GOT, U/L	46.40	56.33	46.40	46.57	6.06	0.51	0.49	0.50
LDH, U/L	498.40	551.33	377.80	387.43	49.59	0.03	0.60	0.72
CPK, U/L	1,974.00^a^	1,901.30^a^	1,925.00^a^	897.10^b^	267.43	0.11	0.10	0.15
GGT, U/L	49.80	52.17	51.20	56.43	4.21	0.58	0.46	0.78
Alb, g/L	39.30	40.30	37.50	39.17	1.26	0.34	0.38	0.82
TP, g/L	75.88	76.67	82.12	81.94	2.60	0.08	0.92	0.88
AMM, μmol/L	88.98	97.60	72.64	87.01	11.87	0.35	0.43	0.84
UN, mmol/L	5.12^a^	4.03^b^	4.47^ab^	5.29^a^	0.28	0.18	0.38	0.05

^a, b, c^ Mean values with unlike superscript letters were significantly different (*P* < 0.05). *n =* 8

GB diets, Chinese conventional diets; B × D, breed × diet interaction; ALP, alkaline phosphatase; GPT, glutamic-pyruvic transaminase; GOT, glutamic-oxaloacetic transaminase; LDH, lactate dehydrogenase; CPK, creatine phosphokinase; GGT, gamma-glutamyl transpeptidase; Alb, albumin; TP, total protein; AMM, blood ammonia; UN, urea nitrogen

In the growing phase, breed × diet interactions were observed (*P* < 0.05) for plasma concentrations of TP and Alb. Plasma activities of ALP and LDH in Landrace pigs were higher (*P* < 0.05) than those in Bama mini-pigs. Landrace pigs fed the GB diet had higher (*P* < 0.05) plasma activities of GPT and GGT, and higher (*P* < 0.05) concentrations of TP and Alb than those fed the NRC diet.

In the finishing phase, no interactions were observed (*P* > 0.05) between breed and diet for any of the plasma biochemical analytes, except for UN (*P* = 0.05). Landrace pigs fed the NRC diet had lower (*P* < 0.05) plasma UN concentration than those fed the GB diet. Bama mini-pigs fed the GB diet had higher (*P* < 0.05) plasma CPK activity than those fed the NRC diet.

## Discussion

The growth performance of pigs and their meat quality depend on the interactive effects of genotype, rearing conditions, pre-slaughter handling, and carcass/meat processing [15–17]. The present study focused on evaluating the effects of breed and dietary protein/energy ratio on growth performance, carcass composition, and meat quality. We found significant interaction effects of breed and diet on growth performance and carcass composition. Bama mini-pigs grew more slowly than Landrace pigs, and their carcasses were composed of less lean meat and more fat than those of Landrace pigs. These findings indicate that there are obvious differences in carcass composition between breeds, as previously reported [18, 19]. Furthermore, the backfat in the native breed was much thicker than that in the Landrace breed. This was expected, as Landrace pigs are the result of several years of genetic selection through Mendelian genetics and molecular genetics approaches for traits including increased growth rate and reduced fat content [20]. Within each breed, pigs fed the NRC diet had considerably higher dressing percentage than those fed the GB diet. However, in Landrace pigs, GB diet promoted carcass length and lean percentage, especially during the growing phase. The accelerated development of bones and muscle may have resulted from compensatory growth due to low nutrition. Landrace pigs fed the NRC diet deposited less fat than Bama mini-pigs. This may result from the high capacity for muscle growth of fast-growing genotypes, such as Landrace pig, which can utilize high nutrient diets without increased lipid deposition.

Meat quality is one of the most important economic traits of farm animals, and it determines the suitability of meat for further processing and storage, including retail display. The main desirable attributes are pH, color, drip loss, fat content, and composition [21]. In our study, Bama mini-pigs exhibited more carcass fat, higher IMF content, and greater backfat thickness, but reduced dressing percentage, lean content of carcass, drip loss, and LD muscle area than did Landrace pigs in the same phase. These findings agree with previous reports of the superior quality of local Chinese pigs [22, 23]. Several studies have shown different dietary protein/energy ratios in animal feeds. Some of these studies examined the growth performance, body composition, and metabolism of aquatic livestock [24], and others were related to obesity and health of humans [25, 26]. Dietary protein/energy ratio has a significant influence on the fat deposition and chemical composition of muscle. Barea et al. [27] reported that gain:feed and gain:metabolizable energy intake were improved by decreasing the ideal CP content of the diet. In their study, when a diet providing 95 g ideal CP per kg DM was fed, protein deposition reached a maximum value of 71 g/day. Hamill et al. [28] reported a two-fold increase in IMF content of the *musculus semimembranosus* of Duroc gilts fed a low-protein diet compared to those fed a high-protein diet. Moreover, they demonstrated, via transcriptome analysis, that regulation of IMF accumulation in response to dietary protein restriction is associated with modulation of gene pathways involved in lipid synthesis and degradation. A high IMF content, also called “marbling fat”, is associated with improved eating quality of meat [29]. The threshold level of IMF in meat that results in a pleasing eating experience is 1.5% IMF, with 2–3 % IMF considered necessary for optimum eating quality [30]. In our study, IMF contents of all muscle samples examined from Bama mini-pigs were greater than 1.5 %. However, the IMF contents varied across the different muscle samples, and IMF contents in BF of Bama mini-pigs differed across phases.

Activities of metabolic enzymes, including ALP, GPT, and GOT, in the blood change in response to the growth and development of animals [31]. Researchers have widely divided opinions on the relationship between ALP, GPT, and GOT and growth performance (especially for ADG) and carcass traits. ALP plays an important role in lipid metabolism; hence, increasing the plasma activity of this enzyme may be useful for promoting ADG [32]. GPT and GOT play a critical role in transamination and reflect the status of protein synthesis and catabolism [33]. An increase in the plasma activities of these enzymes can improve amino acid metabolism. LDH catalyzes the reversible transformation of pyruvate to lactate and plays a principal role in anaerobic cellular metabolism [34]. GGT catalyzes the transfer of the gamma-glutamyl group from glutathione to acceptor amino acids. In this study, plasma activities of ALP and LDH were higher in Landrace pigs than in Bama mini-pigs, especially in the growing phase. Plasma activities of GPT and GGT in Landrace pigs fed the GB diet were higher than those fed the NRC diet. All of these findings were coincident with growth performance within the same phase.

Nitrogen is an indicator of protein status [35] and has been used to determine protein requirements and lean tissue growth rates in pigs. Blood UN, as the ultimate and major nitrogenous product of protein and amino acid catabolism, is synthesized in the body via the ornithine cycle [36, 37]. Plasma UN concentration reflects the balance status of amino acids, and is often used as an indicator of kidney and liver function, as well as an indicator of relative hydration status in animals. Low blood UN indicates a good balance of amino acids, and suggests relatively low urea synthesis and hydration in the liver and relatively high dietary protein efficiency [38]. In the present study, we found that Landrace pigs fed the NRC diet had lower plasma concentration of UN, whereas Bama mini-pigs fed the same diet had higher plasma concentration of UN, than did those fed the GB diet. These findings suggest an interaction effect between breed and diet. In addition, increased protein consumption by mammals results in increased fecal ammonia, which is a polluting substance [39, 40]. Therefore, limiting protein ingestion will also limit the ammonia excretion in fecal substances; lower plasma concentrations of UN could decrease the emission of ammonia from pig production, thereby reducing environmental pollution.

## Conclusions

Compared to Landrace pigs, Bama mini-pigs showed slower growth and lower carcass performance, but had better meat quality, which confirms that this breed of pig is well suited for the production of high-quality pork. Although dietary protein/energy ratio affects the growth performance of Landrace pigs, which depends on the growth stage, we found that the dietary protein/energy ratio did not affect Bama mini-pigs. This finding may be useful for reducing the feed cost and minimizing the adverse effects of ammonia release to the environment in indigenous pig production.

## Additional file

Additional file 1:
**Table S1.** Ingredients and nutrient levels of experimental diets. (DOC 47 kb)

## Abbreviations

ADFI: Average daily feed intake
ADG: Average daily gain
BF: *Biceps femoris*
CP: Crude protein
DM: Dry matter
F/G: Ratio of feed intake to body gain
IMF: Intramuscular fat
LD: *Longissimus dorsi*
PM: *Psoas major*
