# Supplementary material for: Effects of dietary protein/energy ratio on growth performance, carcass trait, meat quality, and plasma metabolites in pigs of different genotypes
Source: J Anim Sci Biotechnol. 2015 Aug 15;6(1):36. doi: 10.1186/s40104-015-0036-x (PMC4537580; doi:10.1186/s40104-015-0036-x)
Supplement: Additional file 1: — Table S1. Ingredients and nutrient levels of experimental diets. (DOC 47 kb) [file 40104_2015_36_MOESM1_ESM.doc]

**Supplementary table 1 Ingredients and nutrient levels of experimental diets**

| Item | NRC diet 1 | NRC diet 2 | NRC diet 3 |  | GB diet 1 | GB diet 2 | GB diet 3 |
| --- | --- | --- | --- | --- | --- | --- | --- |
| Ingredients, % | | | | | | | |
| Corn | 62.80 | 66.00 | 69.50 |  | 63.00 | 60.00 | 66.00 |
| Soybean meal, 42% CP | 26.00 | 28.00 | 23.00 |  | 25.00 | 26.50 | 21.00 |
| Fish meal, 62% CP | 7.00 | 2.00 | - |  | 3.00 | - | - |
| Wheat bran | - | - | 3.00 |  | 6.34 | 10.75 | 10.50 |
| Soybean oil | 1.95 | 1.50 | 2.10 |  | - | - | - |
| CaHPO_4_ | 0.45 | 0.70 | 0.65 |  | 0.80 | 0.80 | 0.50 |
| CaCO_3_ | 0.50 | 0.50 | 0.45 |  | 0.56 | 0.65 | 0.70 |
| Salt | 0.30 | 0.30 | 0.30 |  | 0.30 | 0.30 | 0.30 |
| Premix^1^ | 1.00 | 1.00 | 1.00 |  | 1.00 | 1.00 | 1.00 |
| Nutrient levels | | | | | | | |
| Digestible energy, MJ/kg | 14.22 | 14.21 | 14.22 |  | 13.46 | 13.40 | 13.40 |
| Crude protein^2^, % | 20.06 | 18.01 | 15.11 |  | 18.03 | 16.05 | 13.46 |
| Lys, % | 1.15 | 0.97 | 0.77 |  | 0.96 | 0.86 | 0.74 |
| Met, % | 0.33 | 0.27 | 0.21 |  | 0.27 | 0.23 | 0.21 |
| Thr, % | 0.73 | 0.63 | 0.52 |  | 0.63 | 0.58 | 0.51 |
| Trp, % | 0.23 | 0.21 | 0.18 |  | 0.21 | 0.20 | 0.17 |
| Calcium, % | 0.75 | 0.62 | 0.50 |  | 0.69 | 0.62 | 0.56 |
| Available phosphorus, % | 0.39 | 0.28 | 0.21 |  | 0.21 | 0.13 | 0.12 |

GB diet, Chinese conventional diet.

^1^Premix provided for 1 kg of complete diet: Cu (as Copper sulfate), 10 mg; Fe (as ferrous sulfate), 100 mg; Se (as sodium selenite), 0.30 mg; Zn (as zinc oxide), 100 mg; Mn (as manganese sulfate), 10 mg; V_D3_, 386 IU; V_A_, 3086 IU; V_E_, 15.4 IU; V_K3_, 2.3 mg; V_B2_, 3.9 mg; D-calcium pantothenate, 15.4 mg; nicotinic acid, 23 mg; choline, 80 mg; V_B12_, 0.016 mg.

^2^Crude protein was measured, and other nutrients were calculated.
